# Low-Energy Electron Irradiation Efficiently Inactivates the Gram-Negative Pathogen *Rodentibacter pneumotropicus*—A New Method for the Generation of Bacterial Vaccines with Increased Efficacy

**DOI:** 10.3390/vaccines8010113

**Published:** 2020-03-02

**Authors:** Jasmin Fertey, Lea Bayer, Sophie Kähl, Rukiya M. Haji, Anke Burger-Kentischer, Martin Thoma, Bastian Standfest, Jessy Schönfelder, Javier Portillo Casado, Frank-Holm Rögner, Christoph Georg Baums, Thomas Grunwald, Sebastian Ulbert

**Affiliations:** 1 Fraunhofer Institute for Cell Therapy and Immunology IZI, Perlickstrasse 1, 04103 Leipzig, Germany; jasmin.fertey@izi.fraunhofer.de (J.F.); lea.bayer@izi.fraunhofer.de (L.B.); rukiya.haji@gmail.com (R.M.H.); thomas.grunwald@izi.fraunhofer.de (T.G.); 2 Institute of Bacteriology and Mycology, Centre for Infectious Diseases, Faculty of Veterinary Medicine, University Leipzig, An den Tierkliniken 29, 04103 Leipzig, Germany; sophie.funk@vetmed.uni-leipzig.de (S.K.); Christoph.Baums@vetmed.uni-leipzig.de (C.G.B.); 3 Fraunhofer Institute for Interfacial Engineering and Biotechnology IGB, Nobelstrasse 12, 70569 Stuttgart, Germany; anke.burger-kentischer@igb.fraunhofer.de; 4 Fraunhofer Institute for Manufacturing Engineering and Automation IPA, Nobelstrasse 12, 70569 Stuttgart, Germany; martin.thoma@ipa.fraunhofer.de (M.T.); bastian.standfest@ipa.fraunhofer.de (B.S.); 5 Fraunhofer Institute for Organic Electronics, Electron Beam and Plasma Technology FEP, Winterbergstrasse 28, 01277 Dresden, Germany; jessy.schoenfelder@izi.fraunhofer.de (J.S.); javier.portillo@fep.fraunhofer.de (J.P.C.); frank-holm.roegner@fep.fraunhofer.de (F.-H.R.)

**Keywords:** inactivation of bacteria, low-energy electron irradiation, bacterin, Rodentibacter, electron beam

## Abstract

Bacterial pathogens cause severe infections worldwide in livestock and in humans, and antibiotic resistance further increases the importance of prophylactic vaccines. Inactivated bacterial vaccines (bacterins) are usually produced via incubation of the pathogen with chemicals such as formaldehyde, which is time consuming and may cause loss of immunogenicity due to the modification of structural components. We evaluated low-energy electron irradiation (LEEI) as an alternative method to generate a bacterin. *Rodentibacter pneumotropicus*, an invasive Gram-negative murine pathogen, was inactivated with LEEI and formaldehyde. LEEI resulted in high antigen conservation, and LPS activity was significantly better maintained when compared with formaldehyde treatment. Immunization of mice with LEEI-inactivated *R. pneumotropicus* elicited a strong immune response with no detectable bacterial burden upon sublethal challenge. The results of this study suggest the inactivation of bacteria with LEEI as an alternative, fast and efficient method to generate bacterial vaccines with increased efficacy.

## 1. Introduction

Immunoprophylaxis with inactivated bacteria (bacterins) is widely used in veterinary medicine. Inactivated vaccines are usually produced by incubating the pathogens with chemicals, such as formaldehyde [1], a toxic reagent that inactivates organisms by crosslinking their macromolecules. Although chemical inactivation has been used to generate bacterins for many decades, there are severe drawbacks. In order to obtain full inactivation, pathogens are incubated for days or even weeks with the chemical. The process may also lead to the modification of the pathogen’s surface antigens due to crosslinking, which causes changes in the antigenic properties of the resulting vaccine [2,3]. As a consequence, vaccines may lack critical antigenic epitopes (and might even contain novel unspecific antigens) and their efficacy may be decreased. This problem is well described for a variety of vaccines [4,5,6]. Therefore, the development of novel inactivation procedures that act faster and better preserve the antigenic structures has the potential for generating more efficient and safer vaccines against bacteria. 

As an alternative strategy to chemical treatment, ionizing radiation has been used to generate a variety of experimental bacterial vaccines with promising results [7,8,9,10]. It inactivates by damaging nucleic acids, leaving structural components such as proteins largely intact [11,12]. Compared with using formaldehyde, ionizing radiation has several advantages: it acts much faster, no toxic chemicals are required and the inactivation is more reproducible [13,14]. However, the radiation technologies used so far, i.e., gamma-rays, X-rays or high-energy electron beams, generate high energy radiation that requires complex concrete and lead shielding constructions for its absorption [15,16]. This has so far prevented the use of ionizing radiation for vaccine production. 

In contrast, low-energy electron irradiation (LEEI) only generates low energy X-rays that can be absorbed by a self-shielding design of the equipment with only a few centimeters of lead. This makes LEEI applicable in normal laboratory settings. We have shown previously that LEEI can be used to generate efficient vaccines against viruses and parasites [13,17,18]. To evaluate the potential of LEEI for the development of bacterins with increased protective efficacy, we chose a challenge experiment with *R. pneumotropicus* in its natural host, the mouse. *R. pneumotropicus* is one of the most prevalent infectious agents in laboratory rodents and a member of the *Pasteurellaceae*, which also includes main human and veterinary pathogens such as *Haemophilus influenzae* and *Pasteurella multocida*. Although these Gram-negative bacteria are part of the microbiome of the respiratory tract, they can cause primary or secondary infections, often leading to severe diseases. Experimental infection of wild-type mice with a highly virulent *R. pneumotropicus* strain [19] represents a useful model for improving vaccination strategies against invasive Gram-negative bacteria, even more so as members of the *Pasteurellaceae* exhibit a high degree of host adaptation. We show here that LEEI can be used to inactivate *R. pneumotropicus* and that the irradiated material induces protective immunity. 

## 2. Materials and Methods 

### 2.1. Mice

Female BALB/c mice (6–8 weeks old) were obtained from Charles River (Sulzfeld, Germany) and were kept in a pathogen-free environment in isolated ventilated cages. All animal experiments were carried out in accordance with the EU Directive 2010/63/EU for animal experiments and were approved by local authorities (Landesdirektion Sachsen, No. TVV 07/15; DD24-5131/331/9).

### 2.2. Cultivation of Rodentibacter Pneumotropicus

The *R. pneumotropicus* strain JF4Ni used in this study has been described previously [19]. In brief, bacteria were grown overnight in a Brain Heart Infusion (BHI) medium (Carl Roth) at 37 °C and rotation at 200 rpm. The cells were washed twice in tris-buffered saline (TBS) and diluted to an OD600 nm of 1.0 in TBS before irradiation. To rule out potential contaminations, the genotypes of the challenge strain and of the isolates after challenge were confirmed by PCR using *Rodentibacter*-specific primers as published previously [20]. For challenge experiments, *R. pneumotropicus* was grown to a concentration of 3 × 10^7^ colony forming units (CFU) per milliliter, washed and diluted with phosphate-buffered saline (PBS) before intranasal infection with 6 × 10^6^ CFU/25 µL (infection dose).

### 2.3. Pathogen Inactivation

#### 2.3.1. Low-energy Electron Irradiation (LEEI)

Overnight bacterial cultures were pelleted, washed twice with TBS and diluted to an OD 600 of 1.0. For LEEI treatment, 10 mL of the bacteria solution was filled in disposable PET-PE bags (polyethylenterephthalat-polyethylene 60 µm/20 µm), which were produced using a sealing machine (D 545 AH-2, Kopp Verpackungssysteme, Reichenbach, Germany). The part of the bag to be filled was 31 cm long and 9.2 cm wide. The samples were irradiated with 10, 20 and 30 kGy with a 200 keV electron beam (Linac KeVac System (200 kV/5 mA)) at room temperature. The total irradiation time per bag was 273 s. Doses were calculated based on measurements with a calibrated radiochromic dosimeter film (Risø B3 dosimeter, Risø High Dose Reference Laboratory), on the estimated filled bag´s thickness and on its movement speed through the irradiation area (approx. 1.1 mm per second). After irradiation, the bacterial solution was transferred into 15 mL tubes (Greiner BIO-ONE GmbH) and put on ice until plating and inoculation of liquid cultures. Non-irradiated controls underwent the same procedure except irradiation. Inactivation was confirmed by plating 100 µL of the samples on BHI-agar plates. In parallel, 500 µL of the treated bacterial suspension was added to 5 mL of BHI medium and incubated for 72 h at 37 °C. When no growth was visible during that time, the sample was considered inactivated. The residual samples were stored in aliquots at −80 °C until further use.

#### 2.3.2. Formaldehyde Inactivation

Overnight cultures were pelleted, washed twice with TBS and diluted to an OD 600 nm of 1.0. After the addition of 0.5% (*v*/*v*) formaldehyde (Thermo Scientific, Waltham, MA, USA), bacteria were incubated for 24 h at room temperature. After the procedure the bacteria were washed twice with TBS. Non-irradiated controls underwent the same procedure with addition of PBS instead of formaldehyde. Inactivation was confirmed as described above and residual samples were stored in aliquots at −80 °C until further use.

### 2.4. ELISA

The quality of antigens before and after inactivation was determined by a direct enzyme-linked immunosorbent assay (ELISA). Briefly, wells of a 96-microwell plate were coated with *R. pneumotropicus* (active or inactivated with either LEEI or formaldehyde), diluted in a coating buffer (35 mM Na_2_HCO_3_/15 mM Na_2_CO_3_, pH 9.6) with a total volume of 100 μL per well and incubated at 4 °C overnight. The wells were blocked with PBS containing 5% skim milk. For the detection of antigens, a pool of polyclonal mouse sera drawn from reconvalescent mice experimentally infected with *R. pneumotropicus* strain JF4Ni [19] was used at a 1:800 dilution. The polyclonal sera were added and incubated for 2 h at room temperature. After this incubation, the plates were washed with PBS containing 0.05% Tween 20, and incubated with a 1:5000 dilution of a peroxidase-conjugated rabbit anti-mouse immunoglobulin G antibody (DAKO) at room temperature for 1 h. The plates were then washed three times. TMB-ELISA (BioLegend, San Diego, CA, USA) substrate was used for color development and stopped by addition of 1 M H_2_SO_4_ after 30 min. The absorbance was then determined with a standard ELISA reader at 450 nm and reference wavelength at 520 nm. Each experiment was performed in triplicates. For the analysis of binding antibodies after immunization, active *R. pneumotropicus* was coated as described above, and sera of immunized mice instead of a pool of polyclonal mouse sera were used. All other steps were performed as described above.

### 2.5. LPS Based Reporter Assay

The lipopolysaccharide (LPS) based reporter assay has been previously described [21]. Briefly, serial dilutions of either LEEI- or formaldehyde-inactivated *R. pneumotropicus* were incubated in triplicates with NIH3T3 cells, expressing human TLR4/CD14 together with an inducible alkaline phosphatase under the control of an NF-κB promoter. LPS (3rd Standard *E. coli* O113:H10:K-endotoxin, NIBSC code 10/178) served as a positive control (final concentration of 100 pg/mL). After incubation in a humidified atmosphere for 16 h, activation of the TLR4 pathway resulted in the secretion of alkaline phosphatase into the culture medium. Fifty microliters of the medium were removed and transferred into a 96-well microplate (Greiner BIO-ONE GmbH, Kremsmünster, Austria) and 50 μL of the specific substrate para-nitrophenylphosphate (pNPP, Sigma-Aldrich, St. Louis, MO, USA) was added to each well. Alkaline phosphatase activity was measured by photometrical detection at 405 nm.

### 2.6. Immunization and Challenge

Fifty microliters of LEEI (20 kGy)-irradiated or formaldehyde-inactivated *R. pneumotropicus* containing 2 × 10^7^ cells were mixed with 50 µL 2% Alhydrogel (aluminum hydroxide gel adjuvant, aluminum content 10 mg/mL, Brenntag Nordic A/SSS) per dose. Groups of ten mice each were vaccinated twice at a 4-week intervals by intramuscular administration. Control mice were not immunized. Serum samples were collected from the blood of the animals one week before and three weeks after prime-immunization. Four weeks after the prime, the animals received a boost immunization with the same vaccine preparation or left untreated in the control group. Three weeks after the boost, the animals were bled again for the collection of serum samples. Four weeks after the boost immunization, the mice were challenged intranasally with a sub-lethal dose (6 × 10^6^ CFU) of *R. pneumotropicus* (applied in 25 µL PBS) during a short isoflurane anesthesia. The mice were scored twice daily for signs of disease. The clinical score was assessed by the determination of body weight, the overall appearance, behavior and signs of dyspnea. A total cumulative score of ≥6 for 24 h or a total score of ≥9 at any time point would have been considered a high burden and predefined as termination criteria. As this did not happen, all mice were sacrificed at day 15 post infection with isoflurane pre-anesthesia followed by cervical dislocation and explantation of the lungs.

### 2.7. Determination of Bacterial Load

For the determination of CFU per gram of lung tissue, approximately 100 mg of lung tissue was homogenized in 1 mL PBS, serially diluted and spread in duplicates on Columbia blood (COB) agar plates. Plates were incubated for 48 h at 37 °C and colonies were counted. CFU per gram were calculated and referred to the exact weight in grams of the extracted lung tissue sample.

### 2.8. Statistical Analysis

GraphPad Prism 6 was used to perform statistical analysis. Data was analyzed by *t*-test (unpaired, two-sided), if not stated otherwise. The score after challenge was analyzed using two-way analysis of variance (ANOVA). Bacterial load was analyzed by a Mann–Whitney test. Probabilities lower than 0.05 were considered significant and the *p*-value was indicated by * (* *p* < 0.05; ** *p* ≤ 0.01; *** *p* ≤ 0.001; **** *p* < 0.0001).

## 3. Results

### 3.1. Low-Energy Electron Irradiation Inactivates Bacteria with High Antigen Conservation

**Figure 1:**
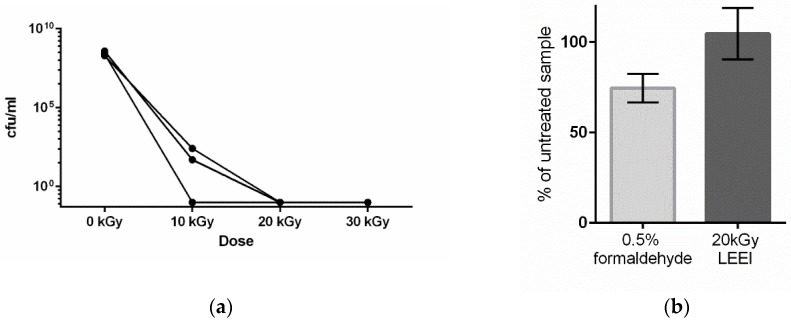
Effect of low-energy electron irradiation (LEEI) on *R. pneumotropicus*. (**a**) *R. pneumotropicus* was irradiated with increasing doses and the resulting colony forming units in the samples were determined. Each curve represents one irradiation experiment with one batch of bacteria. (**b**) Analysis of the conservation of antigenic structures. Equal amounts of LEEI- or formaldehyde-inactivated bacteria were coated on ELISA plates and probed with polyclonal serum from *R. pneumotropicus* infected mice. Measurements with untreated bacteria were used as a reference (set to 100%, error bars represent the standard deviation). The differences are not statistically significant (*t*-test, unpaired, two-sided).

Irradiation experiments were conducted to determine the dose required for the inactivation of *R. pneumotropicus*. Bacterial suspensions were irradiated with 10 kGy, 20 kGy and 30 kGy. While growth was still detectable after the application of 10 kGy, irradiation with 20 kGy and 30 kGy of LEEI reproducibly led to the inactivation of *R. pneumotropicus* (Figure 1a).

In order to compare different inactivation methods, the bacteria were inactivated in two different ways, either by treatment with LEEI at a dose of 20 kGy or by addition of 0.5% formaldehyde and incubation for 24 h. A lower concentration of formaldehyde (0.1%) was tested as well, but resulted in incomplete inactivation (supplementary Figure S1). To determine the antigen conservation of *R. pneumotropicus* after inactivation, treated and untreated bacteria were immobilized on plates and ELISA experiments using a polyclonal serum from previously infected mice were performed. The results showed that after LEEI *R. pneumotropicus* antigens remained fully recognizable (104.7 ± 14.1%) compared with untreated bacteria. Formaldehyde inactivation of the bacteria led to 74.7 % (±7.9%) recognition by the same serum (Figure 1b).

While the chemical inactivation treatment took 24 h, for LEEI inactivation the average handling time for one bag was between 10 and 15 min (including filling, irradiation of the bag and recovery of the sample from the bag).

### 3.2. Low-Energy Electron Irradiation Elicits Strong Immune Responses after Vaccination of Mice

The LEEI- and the formaldehyde-inactivated bacteria were used to immunize mice. The antigen content of both preparations (based on the ELISA measurements in Figure 1b) was equalized, so that both groups received comparable amounts of bacterial antigen.

**Figure 2:**
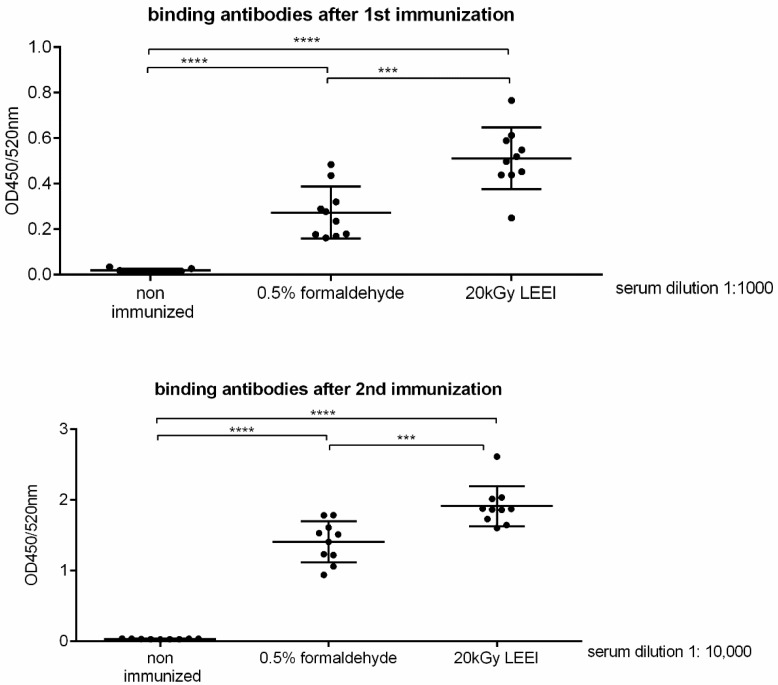
Serum total IgG response in mice after prime and booster vaccinations with bacterins including inactivated bacteria. Ten mice per group were immunized with the two vaccine preparations, while one group of 9 mice remained non-vaccinated. Purified, active *R. pneumotropicus* was coated on ELISA plates and probed with sera from immunized (bacteria inactivated with 20 kGy LEEI or 0.5% formaldehyde, each with alum as adjuvant) and non-immunized mice after the first (prime, upper panel) and second (boost, lower panel) vaccinations. Respective serum dilution is indicated on the right. *P*-value was determined by *t*-test (unpaired, two-sided). *** indicate *p* ≤ significance levels lower than 0.001, **** indicate *p* ≤ significance levels lower than 0.0001. Means and standard deviations are indicated by lines and error bars, respectively.

Mice (ten animals per group) were immunized twice with inactivated bacteria mixed with alum as adjuvant. Significant amounts of *R. pneumotropicus*-specific antibodies were detectable in the animals three weeks after the first immunization (Figure 2). The vaccinated animals in the LEEI group displayed significantly higher antibody titers than the animals that received formaldehyde-treated bacteria. After the second immunization, the titers substantially increased in all animals, and were still significantly higher in the vaccinated animals of the LEEI group than in the corresponding formaldehyde group (Figure 2). No *R. pneumotropicus*-specific antibodies were detected in non-immunized animals at both timepoints.

**Figure 3:**
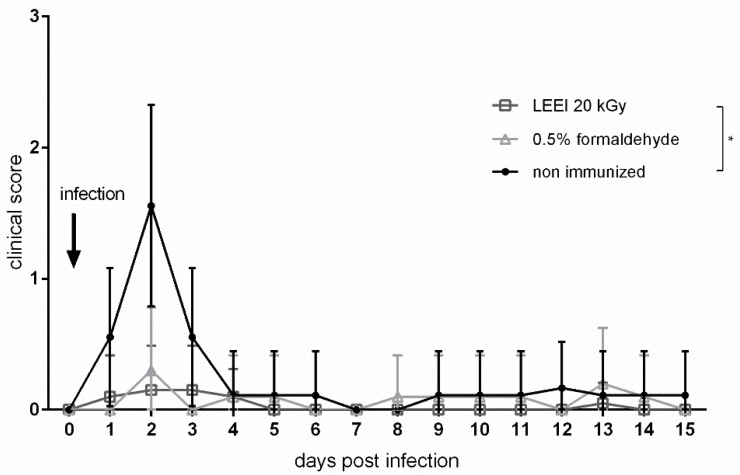
Clinical scores after challenge of vaccinated and non-vaccinated mice. Clinical score of mice after intranasal challenge with a sub-lethal dose of *R. pneumotropicus* strain JF4Ni. Empty grey rectangles indicate LEEI-inactivated material. Empty grey triangles indicate formaldehyde-inactivated material. Black dots indicate non-vaccinated animals (control). A score of 1 was applied to animals losing 5% to 19% of body weight. *p*-value was determined by two-way ANOVA. * indicates *p* ≤ 0.05.

To test whether the immunization with inactivated *R. pneumotropicus* protected against infection of the lower respiratory tract, immunized and control mice were challenged with *R. pneumotropicus* (strain JF4Ni), and the clinical score was analyzed. Due to the high morbidity and mortality observed in previous experiments with this particular strain [19], we decided to use a sub-lethal dose of 6 × 10^6^ CFU per animal. None of the vaccinated mice showed a clinical score above 1 after infection, while five out of nine non-vaccinated animals had a cumulative clinical score of 2, which corresponded to a mean loss of body weight of more than 5%. The mean weight loss was 8% (SD ± 3%), on day 2 post infection. No further specific clinical signs of disease were observed and the mice fully recovered after four days (Figure 3).

**Figure 4:**
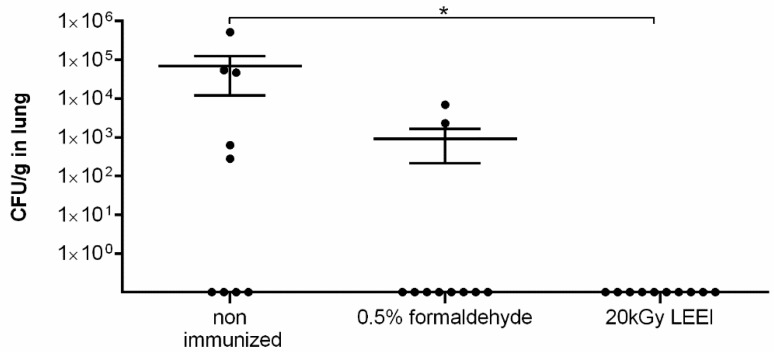
Bacterial load at day 15 post intranasal infection with *R. pneumotropicus* in the lung tissue of vaccinated and control mice. Determination of bacterial load in the lung tissue of challenged mice 15 d.p.i. by investigating colony formation after culturing homogenized lung tissue on COB at 37 °C overnight. Bacteria were inactivated as indicated for preparation of the bacterin. *P*-value was determined by the Mann–Whitney test. * indicates *p* ≤ 0.05. Means and standard deviations are indicated by lines and error bars, respectively.

Fifteen days post-infection mice were euthanized and the bacterial load was determined in the lung tissue. Quantitative analysis revealed that the control mice had on average 7 × 10^4^ CFU/g of lung tissue, while the mice immunized with formaldehyde-inactivated bacteria displayed a (statistically not significant) reduction to a mean value of 9 × 10^2^ CFU/g of lung tissue (Figure 4). In contrast, immunization with LEEI-inactivated bacteria resulted in protection from infection of the lungs as demonstrated by a statistically significant reduction where no cultivatable bacteria were detectable in lung tissue.

**Figure 5:**
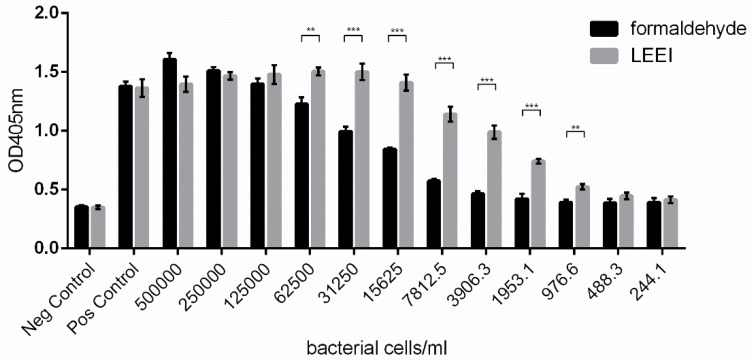
Antigen conservation of the inactivated bacteria used for bacterin vaccination. LPS integrity after inactivation with 20 kGy LEEI or 0.5% formaldehyde was determined by an LPS-reporter assay in which TLR-4 stimulation in NIH3T3 cells led to the secretion of alkaline phosphatase. Shown is one representative measurement of alkaline phosphatase activity after stimulation with dilutions of inactivated *R. pneumotropicus*. Purified LPS served as positive control. Error bars represent the standard deviation (measurement in triplicates). *p*-value was determined by *t*-test (unpaired, two-sided). ** indicates *p* ≤ significance levels lower than 0.01, *** indicates *p* ≤ significance levels lower than 0.001.

Due to the observed differences in the humoral immune response between animals immunized with either LEEI- or formaldehyde-inactivated bacteria, we investigated the quality of the antigenic material in both vaccine preparations by analyzing the LPS functionality. NIH3T3 reporter cells which expressed alkaline phosphatase after TLR4 activation by LPS showed significantly higher expression of the reporter enzyme when incubated with LEEI-inactivated *R. pneumotropicus* than with formaldehyde-inactivated bacteria (Figure 5). This indicated a better conservation of LPS integrity by LEEI as compared with chemical inactivation by formaldehyde.

## 4. Discussion

We have previously shown that LEEI can successfully be used to inactivate a number of pathogens, such as influenza (H3N8), Equid herpesvirus 1, porcine reproductive and respiratory syndrome virus (PRRSV) or respiratory syncytial virus (RSV) [13,17]. Here we report that a bacterin including the LEEI-inactivated Gram-negative pathogen *R. pneumotropicus* elicited a prominent humoral immune response and protection against infection of the lower respiratory tract in mice, the natural host. Vaccines against Gram-negative bacteria are highly relevant in light of the global (re-) emergence of bacterial infections and the increasing resistance to antibiotics. Although *R. pneumotropicus* itself is mainly a problem for laboratory rodents, it is homologous to harmful bacteria of global importance, e.g., *Haemophilus influenzae* and *Pasteurella multocida*—a major human and animal pathogen, respectively, which cause pneumonia or sepsis [22]. It can therefore be assumed that technologies for the protection against *Rodentibacter* will, at least partially, be also applicable to other similar Gram-negative bacteria.

Though bacterial vaccines used in human medicine are mainly conjugated vaccines, bacterins are very important vaccines in veterinary medicine. This includes all autogenous vaccines used in various European countries, which have become very popular as veterinarians are obliged to reduce the use of antibiotics. Based on the results of this study we believe that the protective efficacies of these veterinary bacterins might be substantially improved using LEEI instead of formaldehyde inactivation.

Ionizing radiation has been used to generate bacterial vaccines that elicited protective immune responses in several studies [7,8,9,10,23]. In line with this, our data showed that LEEI has clear advantages over formaldehyde in respect to the conservation of antigenicity in the inactivation process. ELISA and cell-based tests to determine the antigenicity of the inactivated samples showed that inactivation by LEEI was most likely associated with a better antigen conservation and higher LPS integrity than formaldehyde treatment. LPS plays an important role in the immune response by binding to TLR4 and stimulating the innate immunity [24]. There have been attempts to use purified bacterial LPS as a vaccine [25], and it has been investigated as adjuvant in vaccines [26]. The structural integrity of the LPS might therefore be an important factor for stimulating an efficient immune response, especially when using inactivated vaccines. Indeed, in our study, LEEI-inactivated samples led to higher titers of *R. pneumotropicus*-specific antibodies when compared with the formaldehyde treated bacteria.

Inactivation of *Escherichia coli* with (high energy) electron irradiation showed well-preserved membrane integrity. Furthermore, the inactivated *E. coli* cells remained metabolically active up to nine days post-irradiation [27], suggesting that although the nucleic acids were damaged, proteins on the surface remained intact. Though different irradiation techniques were applied in the study by Hieke and Pillai [27] and our work, all results were in accordance with advantageous antigen preservation on the surface of Gram-negative bacteria using irradiation instead of chemical inactivation. A major advantage of LEEI over current radiation technologies used for pathogen inactivation is its applicability in normal laboratories due to the absence of complex shielding constructions.

One limitation of the presented immunization study was the onset of only minor clinical signs induced by the infection. We noticed in a previous study for Balb/c mice that an infection with 10^8^ CFU *R. pneumotropicus* led to the occurrence of clinical signs within 24 h and mortality of over 50% within four days [19]. The approx. 20-fold lower bacterial dose used in this study induced only mild clinical signs such as loss of 8% (SD ± 3%) body weight despite the pronounced bacterial load of the lungs in a few of the non-vaccinated mice.

## 5. Conclusions

The data presented here show that LEEI can be used to generate *R. pneumotropicus* bacterins in a faster and more effective way compared with formaldehyde treatment. LEEI reduces the inactivation time from several hours to minutes and better conserves antigenic structures. Immunization of mice with LEEI-inactivated *R. pneumotropicus* leads to an effective immune response that prevents the infection of the lungs, in contrast to a bacterin based on formaldehyde-inactivated bacteria. LEEI might therefore represent a versatile technique for the generation of vaccines to protect against bacterial infections.

## 6. Patents

M.T. is co-author of a patent application, DE102015224206B3.

## Supplementary Materials

The following are available online at https://www.mdpi.com/2076-393X/8/1/113/s1, Figure S1: Effect of formaldehyde treatment on *R. pneumotropicus*.
