# Supplementary material for: Low-Energy Electron Irradiation Efficiently Inactivates the Gram-Negative Pathogen Rodentibacter pneumotropicus—A New Method for the Generation of Bacterial Vaccines with Increased Efficacy
Source: Vaccines (Basel). 2020 Mar 2;8(1):113. doi: 10.3390/vaccines8010113 (PMC7157226; doi:10.3390/vaccines8010113)
Supplement: Supplementary file 1 [file vaccines-08-00113-s001.pdf]

Supplementary

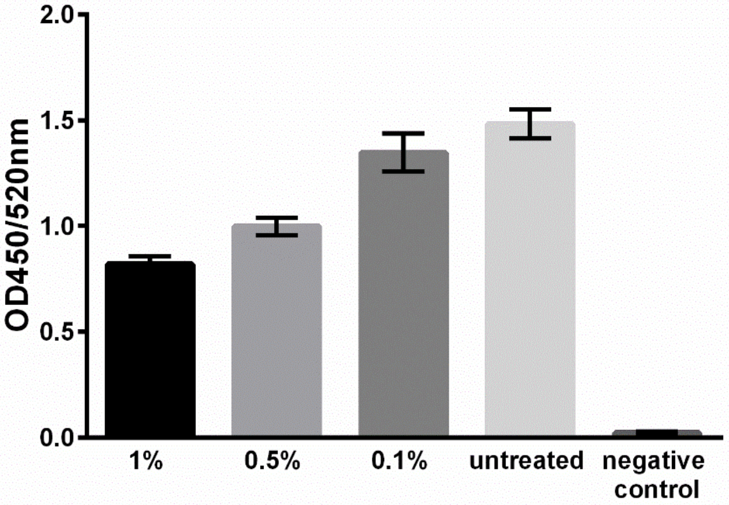

Growth in liquid culture				
Exp. 1	-	-	+	+
Exp. 2	-	-	-	+

Figure S1. Effect of formaldehyde treatment on *R. pneumotropicus.*

Overnight cultures of *R. pneumotropicus* were washed with TBS and treated with different formaldehyde concentrations ranging from 0.1 % to 1 % for 24 h at room temperature. After washing with TBS, residual infectivity was tested in liquid culture. Tabular results of two independent experiments are shown below the graph. For analysis of the conservation of antigenic structures, equal amounts of bacteria were coated on ELISA plates and probed with polyclonal serum from *R. pneumotropicus* infected mice. Bars represent mean values of the two experiments, error bars show the standard deviation. TBS served as negative control.
